# Comparative efficacy of unilateral biportal endoscopy and micro-endoscopic discectomy in the treatment of elderly patients with lumbar spinal stenosis

**DOI:** 10.12669/pjms.42.5.15073

**Published:** 2026-05

**Authors:** Renbo Li, Bo Chen, Xin Ai, Guang Yang, Wei Zhou

**Affiliations:** 1 Renbo Li, Second Department of Spine Surgery, Dalian Third People’s Hospital, Dalian, Liaoning Province 116091, P.R. China; 2 Bo Chen, Second Department of Spine Surgery, Dalian Third People’s Hospital, Dalian, Liaoning Province 116091, P.R. China; 3 Xin Ai, Second Department of Spine Surgery, Dalian Third People’s Hospital, Dalian, Liaoning Province 116091, P.R. China; 4 Guang Yang, Second Department of Spine Surgery, Dalian Third People’s Hospital, Dalian, Liaoning Province 116091, P.R. China; 5 Wei Zhou, Second Department of Spine Surgery, Dalian Third People’s Hospital, Dalian, Liaoning Province 116091, P.R. China

**Keywords:** Clinical efficacy, Elderly patients, Lumbar spinal stenosis, Micro-endoscopic discectomy, Unilateral biportal endoscopy

## Abstract

**Objective:**

Currently, the comparative clinical efficacy of unilateral biportal endoscopy (UBE) and microendoscopic discectomy (MED) in the treatment of elderly lumbar spinal stenosis (LSS) is unclear. This study compared the clinical advantages of UBE and MED.

**Methodology:**

This retrospective study was conducted at Dalian Third People’s Hospital and included clinical data of 105 elderly LSS patients who underwent surgical treatment from March 2023 to July 2024. According to different surgical methods, patients were divided into the UBE group (n=48) and the MED group (n=57). The study compared baseline characteristics, perioperative indicators, and clinical outcomes at three days, one month, six months, and one year post procedure. Lower back and leg pain was assessed using the Visual Analog Scale (VAS) and the Oswestry Disability Index (ODI).

**Results:**

UBE was associated with a considerably shorter length of surgery, less intraoperative blood loss, and a shorter hospital stay (P < 0.05). There were no statistically significant differences in the VAS and ODI scores between the UBE and MED groups (P > 0.05). VAS scores and ODI scores markedly improved in both groups at all postoperative time points (P < 0.05). UBE resulted in a significantly lower overall complication rate than MED (P<0.05).

**Conclusions:**

UBE and MED demonstrate comparable clinical efficacy in relieving pain and improving function in elderly patients with LSS. However, UBE exhibits significant advantages in perioperative safety and overall complication rates.

## INTRODUCTION

Lumbar spinal stenosis (LSS) is a degenerative spinal disease that is prevalent in the elderly and is characterized by reduced lumbar spinal volume and compression of neural tissues.^1^,^2^ With the gradual aging of the population, the incidence of LSS is currently on the rise.^3^,^4^ The prevalence of LSS is approximately 10-20% in adults > 60 years old, and the prevalence increases by 5-8% for every 10-year increase in age.^2^–^4^ LSS often presents with intermittent claudication, low back and leg pain, and sensory and motor disorders of lower extremities, which seriously affect patients’ daily activity ability and quality of life.^5^

Currently, the management of LSS primarily consists of conservative and surgical treatments.^6^–^9^ Conservative treatment includes analgesic medication, rehabilitation exercises, and brace fixation.^6^ However, more than 40% of patients will still require surgical intervention due to progressive aggravation of nerve compression.^6^,^7^ With the development of medical technology, minimally invasive and endoscopic techniques have been widely applied in spinal surgery.^7^,^8^ Micro-endoscopic discectomy (MED) is currently the preferred minimally invasive method for treating LSS; compared with traditional open surgery, it has advantages such as less trauma, less blood loss, and faster recovery.^9^ Nevertheless, MED has limitations, including a restricted surgical field of view and incomplete decompression.^9^,^10^

Recently, a novel minimally invasive lumbar spine surgery, unilateral biportal endoscopy (UBE), utilizing two independent portals (one for the endoscope and the other for introducing surgical instruments) has become popular in clinical practice due to the biportal field of view and flexible operating space.^11^,^12^ Both UBE and MED are practical approaches for treating single-segment LSS in elderly patients.^12^ However, limited studies have compared the advantages and disadvantages of these two methods. This retrospective study aimed to clarify the benefits and limitations of UBE and MED in the treatment of single-segment LSS in elderly patients.

## METHODOLOGY

Clinical data of 105 elderly patients with LSS who underwent surgical treatment at Dalian Third People’s Hospital from March 2023 to July 2024 were retrospectively analyzed. According to the different surgical methods, the patients were divided into the UBE and the MED group.

### Ethical approval:

The study was approved by the ethics committee of the Dalian Third People’s Hospital (Approval # 2025-214-001; dated: October 15, 2025). Due to the study’s retrospective design, patient consent was waived.

### Inclusion criteria:

- Single-segment LSS confirmed by computed tomography (CT) or magnetic resonance imaging (MRI) examination.^1^
- Age ≥ 70 years.
- Low back pain or radicular leg pain with or without intermittent neurogenic claudication.
- No significant improvement in symptoms despite standardized conservative treatment.
- Follow-up period ≥ 12 months.

### Exclusion criteria:

- Spinal tumors, severe spinal deformities, or spondylolisthesis (≥ Grade II).
- Recurrent LSS.
- Cauda equina syndrome.
- Lumbar spine fractures.
- Severe systemic diseases or mental illnesses.

### Perioperative management and surgical approach:

All patients underwent standardized general anesthesia. For patients taking anticoagulants or antiplatelet medications, a uniform perioperative protocol was strictly followed, requiring the discontinuation of these medications for 7 to 10 days prior to surgery.

### UBE and MED procedures:

Following general anesthesia and prone positioning, target segments were localized using C-arm fluoroscopy. For the UBE group, two independent portals (viewing and working) were established via 0.7-1.0 cm incisions to perform targeted laminectomy, flavectomy, and nerve root decompression. For the MED group, a single 1.5-2.0 cm paramedian incision was made to dock a tubular retractor for subsequent microscopic decompression. In both groups, adequate bilateral decompression was achieved when indicated, followed by rigorous hemostasis, placement of a negative-pressure drain, and layered closure. For a highly granular and standardized description of the operative workflows for both UBE and MED—including precise portal placement, bony decompression extent, ligamentum flavum handling, the unilateral approach for bilateral decompression technique, energy device utilization, and standardized drain criteria—please refer to the Supplementary Technical Note.

### Data collection:

The following information was collected from all patients:

- General information, including gender, age, body mass index (BMI), smoking status, lesional segment, stenosis type, affected side, and American Society of Anesthesiologists (ASA) classification.
- Perioperative data, including operation duration, intraoperative blood loss (IBL), number of intraoperative fluoroscopies, and length of hospital stay.
- *Primary endpoint:* The primary endpoint was the improvement in the Oswestry Disability Index (ODI) at the one-year postoperative follow-up. The ODI was used to evaluate the improvement of lumbar spine function; it ranges from 0 to 50 and is calculated as ODI = (actual score / 50) × 100%, with higher scores indicating greater disability.
- *Secondary endpoints:* The secondary endpoints included the Visual Analog Scale (VAS) scores for low back and leg pain at three days, one month, six months, and one year post-procedure; perioperative indicators (operation duration, intraoperative blood loss, length of hospital stay); and the overall complication rate. The VAS ranges from 0 to 10, with higher scores indicating more severe pain.
- *Complications and severity grading:* Complications were strictly defined and recorded, including dural tear (confirmed via direct intraoperative visualization), paraspinal muscle injury (diagnosed based on persistent localized postoperative pain and tenderness requiring extended physical therapy), spinal epidural hematoma (defined as clinically significant events presenting with new-onset nerve root irritation, rather than incidental imaging findings), and wound infection (diagnosed according to the Centers for Disease Control and Prevention [CDC] criteria for superficial surgical site infections). The severity of all complications was graded using the Clavien-Dindo classification system, and the need for re-intervention or reoperation was documented.

### Statistical analysis:

SPSS version 27.0 (IBM, Armonk, NY, USA) was used for all analyses. Categorical variables were presented as absolute numbers and percentages, and their associations were analyzed using the chi-square test. Visual (histograms and Q-Q plots) and analytical (Kolmogorov-Smirnov/Shapiro-Wilk tests) methods were employed to determine whether the variables followed a normal distribution. Continuous variables with a normal distribution were expressed as mean ± standard deviation (SD); Comparisons between the two groups were conducted using the independent samples t-test, and for repeated-measures data, repeated-measures analysis of variance (ANOVA) was used for intragroup comparisons across different time points. For variables that did not follow a normal distribution, data were described as median with interquartile range (IQR); Comparisons between the two groups were performed using the Mann-Whitney U test, and for repeated-measures data, the Friedman test was used for intragroup comparisons across different time points. A P-value < 0.05 was considered statistically significant. PRISM 8.0 software (GraphPad, San Diego, USA) was used to generate line graphs showing changes in VAS and ODI scores at different time points.

## RESULTS

After screening based on the inclusion and exclusion criteria, 105 patients (60 males and 45 females) were enrolled in the study. Ages ranged from 70 to 89 years, with a median age of 76 (73–80) years. There were 48 patients in the UBE group and 57 patients in the MED group, with no statistically significant differences in baseline data between the two groups (P>0.05) (Table-I).

**Table-I:** Comparison of baseline data between two groups.

Baseline	UBE group (n=48)	MED group(n=57)	χ^2^/t	P
Gender, n(%)			1.036	0.309
Male	30 (62.5)	30 (52.6)		
Female	18 (37.5)	27 (47.4)		
Age (year), M(IQR)	75 (72.5-79.5)	76 (74-80)	-	0.086
BMI (kg/m²), Mean±SD	22.9±2.8	22.7±2.9	0.283	0.778
Smoking status, n(%)	23 (47.9)	27 (47.4)	0.003	0.955
Lesional segment, n(%)			3.041	0.219
L3-L4	3 (6.3)	1 (1.8)		
L4-L5	25 (52.1)	24 (42.1)		
L5-S1	20 (41.7)	32 (56.1)		
Stenosis type, n(%)			3.992	0.136
Central Lumbar Spinal Stenosis	17(35.4)	12 (21.1)		
Lateral Recess Stenosis	22 (45.8)	37 (64.9)		
Nerve Root Canal Stenosis	9 (18.8)	8 (14.0)		
Stenosis side, n(%)			1.512	0.219
Unilateral	30 (62.5)	42 (73.7)		
Bilateral	18 (37.5)	15 (26.3)		
ASA classification, n(%)			1.636	0.441
I	3 (6.3)	2 (3.5)		
II	39 (81.3)	43 (75.4)		
III	6 (12.5)	12 (21.1)		

As shown in Table-II, the duration of surgery, intraoperative blood loss, and length of hospital stay in the UBE group were significantly lower than those in the MED group (P<0.05); However, there was no statistically significant difference in the number of intraoperative fluoroscopies between the two groups (P>0.05).

**Table-II:** Comparison of perioperative indicators between the two groups.

Index	UBE group (n=48)	MED group (n=57)	t	P
Duration of surgery (min)	80.6±16.7	90.5±19.3	-2.798	0.006
Intraoperative blood loss (mL)	85.1±22.5	96.4±28.6	-2.248	0.027
Number of intraoperative fluoroscopies (time)	5.98±2.28	6.91±3.02	-1.801	0.075
Length of hospital stay (day)	7 (5-10)	9 (7-11)		0.022

**Fig.1:**
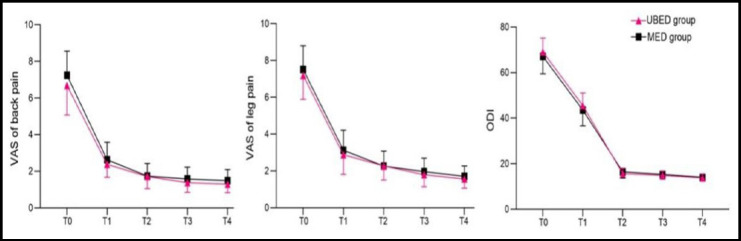
(A) Changes in low back pain VAS scores over time. (B) Changes in leg pain VAS scores over time. (C) The curve of ODI score over time. T0, before surgery; T1, three days postoperatively; T2, one month postoperatively; T3, six months postoperatively; T4, one year postoperatively.

As shown in Fig.1, compared with the preoperative scores, the VAS scores and ODI scores in both groups decreased significantly after surgery (P < 0.05). There were no statistically significant differences in VAS scores and ODI scores between the two groups before surgery, as well as at three days, one month, six months, and one year after surgery (P > 0.05).

The observed complications in the UBE group included three cases of dural tear, one case of spinal epidural hematoma, and one case of wound infection (Table-III). In the MED group, there were three cases of dural tear, five cases of paraspinal muscle injury, four cases of spinal epidural hematoma, and four cases of wound infection (Table-III). The overall complication rate in the UBE group (10.42%) was significantly lower than that in the MED group (28.07%) (P < 0.05).

**Table-III:** Comparison of complications between the two groups.

Complications	UBE group (n=48)	MED group (n=57)	P
Dural tear	3 (6.25)	3 (5.26)	
Paraspinal muscle injury	0 (0.00)	5 (8.77)	
Spinal epidural hematoma	1 (2.08)	4 (7.02)	
Wound infection	1 (2.08)	4 (7.02)	
Total	5 (10.42)	16 (28.07)	0.039

## DISCUSSION

This study retrospectively compared the efficacy and safety of UBE and MED in the treatment of LSS. One year after the surgery, both methods demonstrated comparable clinical efficacy in terms of pain relief and functional improvement. However, UBE was associated with a significantly shorter duration of surgery, less intraoperative blood loss, a shorter length of hospital stay, and a lower overall complication rate. It is crucial to distinguish statistical significance from clinical relevance in this context. While the minor reductions in operative time and blood loss reached statistical significance, the most clinically relevant finding for the elderly population is the substantial reduction in the overall complication rate (10.42% vs. 28.07%). For fragile geriatric patients with limited physiological reserves, minimizing complications such as epidural hematoma or wound infection is of paramount clinical value for ensuring surgical safety and accelerating recovery. These results are consistent with previous studies.^12^–^14^

In terms of perioperative outcomes, the UBE group experienced shorter operation durations, less intraoperative blood loss, and a shorter hospital stay, which confirms observations of Feng et al.^13^ A study by Zhang et al.^14^ indicated that while both UBE and MED bilateral decompression could significantly improve LSS symptoms, UBE was associated with shorter operation time, lower surgical complication rate, fewer intraoperative X-ray fluoroscopies, earlier first ambulation time and shorter hospital stay after surgery. This difference between UBE and MED may be due to the dual-channel operation system of UBE, which enables the use of arthroscopes and conventional surgical instruments without being limited by specialized instruments.^11^,^12^ Compared with MED, UBE allows surgeons to operate more freely with fewer restrictions, facilitating direct manipulation of instruments.^13^–^15^ This dual-channel function has significant advantages in surgeries such as intervertebral disc nucleotomy, spinal canal decompression, and intervertebral fusion.^15^ However, surprisingly, this study showed that the number of intraoperative fluoroscopies was similar in the UBE and the MED groups. This result may be related to the experience of the surgeons performing the procedures.

In terms of pain relief and functional improvement, there were no significant differences in VAS scores and ODI scores between the two groups at all time points. This suggests that both UBE and MED can achieve effective decompression. From a biological perspective in an elderly cohort, while UBE offers advantages in perioperative safety and tissue preservation, the fundamental objective of both procedures remains the alleviation of neural compression. Once thorough decompression is achieved, the long-term functional recovery trajectories tend to converge due to the biological limitations of nerve regeneration in the elderly and the shared endpoint of decompression. These results are consistent with previous reports.^13^–^16^ Although UBE involves more surgical steps, it follows the typical workflow of open decompression, enabling adequate removal of hypertrophied ligamentum flavum and decompression of the lateral recess and nerve root, thereby reducing residual neural compression.^15^,^16^ Although MED uses a single channel, it can accurately reach the stenotic segment through a paramedian approach for nerve root decompression.^17^ The results of this study demonstrate that both surgical methods can achieve adequate decompression, leading to satisfactory pain relief and functional recovery. However, a meta-analysis by Meng et al.^18^ showed that UBE was more effective in relieving low back and leg pain in LSS patients. In addition, a study by Aygun et al.,^19^ which included 154 LSS patients, found that UBE is associated with lower ODI scores at multiple postoperative time points after two years of follow-up. It is plausible that this difference may be related to the more extensive damage to paravertebral soft tissues during MED surgery.

UBE in this study was associated with a significantly lower complication rate than MED (10.42% vs 28.07%, respectively). While both groups experienced perioperative complications such as dural tears, epidural hematomas, and wound infections, paraspinal muscle injuries were exclusively observed in the MED group. For dural tears, no specialized repair was performed due to their small size and the absence of symptomatic cerebrospinal fluid leakage; they were managed with standardized conservative observation, including increased postoperative bed rest.^19^,^20^ For paraspinal muscle injury, detumescence, pain relief, and rehabilitation physical therapy were administered. For clinically symptomatic spinal epidural hematoma, drugs to improve microcirculation were given intravenously, resulting in complete symptom resolution without surgical intervention. For wound infection, local debridement and oral antibiotics were used successfully, with no progression to deep tissue infection.^21^ Consequently, all observed complications in this study were minor and categorized as Grade I or II according to the Clavien-Dindo classification. Most importantly, the postoperative reoperation rate for both the UBE and MED groups was 0%. The observed lower complication rate of UBE can be more comprehensively understood through an elderly-specific explanatory framework. Elderly LSS patients often present with diminished physiological reserve, sarcopenia, osteoporosis, and complex degenerative anatomy such as severely hypertrophic ligamentum flavum and distorted spinal canal structures. Under these challenging conditions, the unique dual-portal system of UBE provides a “biportal field of view” that far exceeds the restricted visualization of MED. The independent viewing portal and flexible operating space allow surgeons to navigate the complex degenerative anatomy with reduced technical difficulty. This superior visualization and instrument flexibility play a central role in minimizing intraoperative blind spots, thereby significantly lowering the incidence of paraspinal muscle injury and epidural hematoma—complications that are particularly detrimental to fragile elderly tissues. Furthermore, by facilitating precise manipulation, UBE achieves more thorough decompression while minimizing surgical trauma, reducing the occurrence of iatrogenic complications in this vulnerable population.^18^,^20^,^22^,^23^

### Strengths of the study:

First, it specifically focuses on a vulnerable geriatric cohort aged 70 years and older. Given the diminished physiological reserves and complex degenerative spinal anatomy characteristic of this population, our direct comparison between UBE and MED provides highly relevant and much-needed safety evidence for clinical decision-making. Second, all surgical procedures were performed by a single, highly experienced team, which minimized technical heterogeneity and learning curve biases. Consequently, our finding that UBE significantly reduces perioperative complication rates provides compelling, standardized evidence for optimizing surgical outcomes in fragile elderly patients.

### Limitations:

First, the retrospective, non-randomized design may introduce selection bias, and relying solely on P-values cannot guarantee absolute baseline comparability. Additionally, the small sample size and limited complication events precluded advanced confounding adjustments like Propensity Score Matching (PSM) or multivariable regression to avoid unstable estimates. Second, historical records lacked standardized quantitative data on key clinical and imaging characteristics, including stenosis severity, osteoporosis degree, subtle instability, and facet preservation. This prevents full adjustment for unmeasured confounders and limits the comprehensive evaluation of technical difficulty. Third, relying on subjective VAS and ODI scores may not fully capture the multidimensional surgical benefits in the elderly. Essential patient-centered outcomes, including walking distance, analgesic use, and quality-of-life metrics like SF-36 or EQ-5D, were not systematically recorded, potentially underestimating true functional recovery. Fourth, surgeon-specific factors and learning curves could influence perioperative metrics. Finally, as this study only included elderly patients with single-segment LSS, generalizing these results to broader LSS populations warrants caution.

SUPPLEMENTARY TECHNICAL NOTE: STANDARDIZED OPERATIVE WORKFLOWS

**1. Unilateral Biportal Endoscopy (UBE) Technique**

***Patient Positioning and Localization:***

Following the induction of general anesthesia, the patient is placed in a prone position on a radiolucent Wilson frame to reduce abdominal pressure and widen the interlaminar space. The targeted surgical segment is precisely identified and marked using intraoperative C-arm fluoroscopy.

***Incision and Portal Placement:***

Two longitudinal skin incisions are made along the medial pedicle line of the targeted level. The viewing portal (approximately 0.7 cm in length) is established 1.5 cm cranial to the targeted intervertebral disc space, while the working portal (approximately 1.0 cm in length) is created 1.5 cm caudal to the disc space. Sequential dilators are used to bluntly separate the paraspinal muscles, and a continuous saline irrigation system (gravity-assisted) is established to maintain a clear visual field and control minor epidural venous bleeding.

***Instrumentation and Energy Devices:***

A high-speed diamond burr (usually 3.5 mm or 4.0 mm in diameter) is utilized for precise bone milling. Soft tissue ablation, continuous clearing of the visual field, and meticulous hemostasis are strictly managed using a radiofrequency (RF) plasma wand (e.g., ArthroCare). This specific energy device provides controlled coagulation while minimizing thermal spread to the adjacent dural sac and nerve roots.

***Bony Decompression and Ligamentum Flavum Handling:***

Under continuous endoscopic visualization (usually using a 0° or 30° endoscope), an ipsilateral partial laminectomy is performed. The surgeon resects the inferior margin of the cranial lamina and the superior margin of the caudal lamina using the diamond burr. The medial aspect of the inferior articular process is carefully undercut to achieve lateral recess decompression while preserving the mechanical stability of the facet joint. The hypertrophied ligamentum flavum is detached from its cranial, caudal, and lateral bony attachments. It is then removed either en bloc or piecemeal using Kerrison rongeurs to expose the ipsilateral traversing nerve root and the dural sac.

***Unilateral Approach for Bilateral Decompression (ULBD):***

For patients requiring bilateral decompression, the “over-the-top” technique is employed. The base of the spinous process and the ventral cortex of the contralateral lamina are undercut with the burr. The contralateral ligamentum flavum is subsequently detached and resected using Kerrison rongeurs to safely decompress the contralateral traversing nerve root and lateral recess, crucially without compromising the structural integrity of the contralateral facet joint or pars interarticularis.

***Drain Criteria and Closure:***

Upon confirmation of adequate decompression (evidenced by the free pulsation and mobility of the bilateral nerve roots) and meticulous hemostasis, a negative-pressure drainage tube is routinely inserted into the epidural space through the working portal. The incisions are closed in layers. The drain is standardized to be removed when the output is less than 30 mL/24h, or within 48 hours postoperatively.

***2. Micro-endoscopic Discectomy (MED) Technique***

***Patient Positioning and Localization:***

The patient positioning and fluoroscopic localization procedures are identical to those described for the UBE group.

***Incision and Portal Placement:***

A single 1.5–2.0 cm paramedian longitudinal incision is made approximately 1.5 cm lateral to the spinous process of the targeted intervertebral level. Serial tubular dilators are sequentially inserted through the lumbodorsal fascia and paraspinal musculature to dock a fixed tubular retractor (typically 18–22 mm in diameter) firmly on the ipsilateral lamina-facet junction.

***Instrumentation and Energy Devices:***

Within the confined space of the tubular retractor, bipolar electrocautery is the primary modality utilized for soft tissue coagulation and hemostasis, as opposed to the continuous saline irrigation and RF plasma used in UBE.

***Bony Decompression and Ligamentum Flavum Handling:***

Under microscopic or fixed-endoscopic visualization, specialized long-handled osteotomes, high-speed burrs, and Kerrison rongeurs are used to resect the medial portion of the inferior articular process and perform an ipsilateral laminotomy. The exposed ligamentum flavum is grasped with forceps, incised, and resected to safely access the epidural space and identify the compromised nerve root.

***Unilateral Approach for Bilateral Decompression:***

To achieve bilateral decompression through the single, fixed tube, the tubular retractor is “wanded” or angled medially toward the midline. The surgeon undercuts the base of the spinous process and the contralateral lamina using angled curettes and Kerrison rongeurs to meticulously remove the contralateral ligamentum flavum. This specific process demands significant technical maneuvering and spatial awareness, as the visualization angle and instrument mobility are inherently more restricted compared to the independent, unconstrained portals of the UBE system.

***Drain Criteria and Closure:***

Following the confirmation of nerve root release and thorough hemostasis, a negative-pressure drain is placed through the tubular corridor. The fascial layer and skin are sutured. Identical to the UBE cohort, the drain is removed when the drainage volume falls below 30 mL/24h.

## CONCLUSION

This study indicates that UBE and MED provide comparable clinical efficacy in terms of pain relief and functional improvement for elderly patients with LSS. However, UBE is associated with significant advantages in secondary endpoints, including a shorter operation duration, less intraoperative blood loss, a shorter length of hospital stay, and most importantly, a substantially lower complication rate. Further multicenter, large-sample, and long-term follow-up randomized controlled trials are needed to provide more robust evidence.

### Suggestions:

To overcome the inherent limitations of this single-center retrospective design, there is an urgent need for multicenter, large-scale randomized controlled trials (RCTs). Furthermore, future studies should integrate multidimensional evaluations to provide more robust clinical guidelines. This includes incorporating patient-centered, long-term quality-of-life metrics (such as the SF-36 or EQ-5D), conducting thorough cost-effectiveness analyses, and utilizing precise, imaging-based objective evaluations of decompression adequacy.

### Author’s contributions:

**RL BC:** Literature search, study design and manuscript writing.

**XA, GY and WZ:** Data collection, data analysis and interpretation. Critical Review.

**RL and BC:** Manuscript revision and validation and is responsible for the integrity of the study.

All authors have read and approved the final manuscript.
